# Prevalence of Hepatitis B Virus infection and associated factors among female sex workers using respondent-driven sampling in Hawassa City, Southern Ethiopia

**DOI:** 10.1186/s12866-022-02444-x

**Published:** 2022-01-31

**Authors:** Deresse Daka, Getahun Hailemeskel, Demissie Assegu Fenta

**Affiliations:** 1 School of Medical Laboratory Science, College of Medicine and Health Science, Hawassa University, Hawassa, Ethiopia; 2 Department of Laboratory Hawassa University Comprehensive Specialized Hospital, Hawassa, Ethiopia

**Keywords:** Hepatitis B virus, Female sex workers, Hawassa, Ethiopia

## Abstract

**Background:**

Female sex workers (FSWs) are a marginalized group notoriously having limited healthcare access and poor-quality care. Inevitably, they are vulnerable to sexually transmitted infections including hepatitis B virus. However; Hepatitis B virus infection is one of the most serious infections and major public health problem considered to be at soaring risk for transmission and acquisition of the infection. Hence, this study was aimed to assess the prevalence and associated factors of HBV infections among FSWs in southern Ethiopia.

**Methods:**

A cross-sectional study was conducted from November to February 2019 at Hawassa city in ISHDO confidential clinic among 383 FSWs. Respondent-driven *consecutive* sampling was used to select study participants using a standardized questionnaire. Blood sample was collected and viral surface antigen was detected using ELISA from separated serum. Data were entered to SPSS version 21.0. Descriptive and logistic regression analyses were used.

**Result:**

The overall prevalence of FSWs who were tested for HBV using ELISA was 35(9.2%) (95% CI: 6.3–12.1). Among 381 FSWs 249(65.4%) were stayed for 2–5 years in sexual work and 240(63%) of them were used condom consistently during sexual practice. In multivariate logistic regression analysis, FSWs who didn’t use condom were six and two times more risk full to acquire HBV than those who used condom commonly (AOR = 6.38, CI 2.04–18.51) and condom breakage (AOR = 2.10, CI 1.95–4.65), during sexual practice respectively. Similarly, use of stimulants (AOR = 3.25, CI 1.59–18.63), previous history of STI (AOR = 2.15, CI 1.02–6.93), genital ulcer (AOR = 4.64, CI 1.31–11.35), number of sexual partners (AOR = 3.25, CI 1.59–7.47), sex during menses (AOR = 5.85, CI (1.29–21.44), sexual assault (AOR = 2.93, CI 1.23–9.01), sharp material sharing, (AOR = 4.98, CI 1.34–10.95) and history of abortion, (AOR = 2.46, CI 1.18, 12.19), were statistically associated with HBV infection. Factors such as age, residence, and alcohol consumption were not associated with HBV infection.

**Conclusion:**

The prevalence of HBV infection in this study was relatively high compared to the general population. Factors like sociodemographic, behavioral, and previous history-related information were associated with HBV infection shows the need for ongoing screening of high-risk population to inform planning for vaccination and preventive measures.

## Background

Hepatitis B virus (HBV) is a DNA virus belonging to the Hepadnaviridae family [1, 2]. Despite the availability of a safe and effective vaccine against hepatitis B infection for over two decades now, the overall burden of the disease remains enormous with over two billion people infected worldwide and approximately one million deaths occur annually from HBV-related illness [1, 3]. According to the WHO report over 240 and 150 million populations were infected with chronic liver disease due to HBV and HCV respectively [1].

Hepatitis B virus is endemic in sub-Saharan Africa, and despite the introduction of universal hepatitis B vaccination and effective antiviral therapy, the estimated overall seroprevalence of hepatitis B surface antigen remains high at 6·1% [4].

Hepatitis B virus prevalence is highest in an adult population of the Western Pacific Region and Africa, where 6.2 and 6.1% respectively [4, 5]. The prevalence of HBV infection among the general population of the Eastern Mediterranean Region, South-East Asia, Europe, and Americas, was 3.3, 2.0, 1.6%, and 0.7 respectively [5]. It is mentioned that Africa is one of the continents with the highest prevalence of HBV and Ethiopia being a member of these continents and shares the burden [1].

Hepatitis B virus is usually transmitted through exposure to infected blood and various body fluids (saliva, menstrual, vaginal, and seminal fluids) and recycle of needles and syringes either in healthcare settings or along with persons who inject drugs. Furthermore; the infection can arise during medical, surgical, and dental procedures, through tattooing, or through the utilization of razors and related objects that are contaminated with infected blood [5].

The prevalence of HBV among female sex workers in different studies conducted in African countries revealed that 4.2% in the Republic of Congo [6], 17.1% in Nigeria [7], 18.2% Burkina Faso [8], 4% in South Africa [9], and 2.5% in Ruanda [10]. From limited studies in Ethiopia, the highest prevalence was reported from Gondar 28.9% [11], Mekelle 6% [12], and Dessie 13.1% [13]. In other developed countries such as Brazil [14], China [15], Thailand [16], and India [17] the prevalence of HBV among Female sex workers was 23.6, 10.7, 11.4, and 3.6% respectively.

Different factors such as workplace associated factors, inconsistent condom use, substance, and drug use [12] economic factors, marital interruption, low educational status, and unsafe alcohol use, and co-infection with other sexually transmitted infections were factors associated with HBV infection [9].

Sex work remains an important contributor to HBV and HIV transmission within early, advanced, and regressing epidemics in sub-Saharan Africa, and its social and behavioral factors play an important role in the transmission of these diseases [5]. Female sex workers (FSW) are more prone to HBV and other sexually transmitted infections (STIs) as well as transmitting them to the public through their clients as they are often in a poor position to negotiate safe sex because of social, economic, cultural, and legal factors [18].

In sub-Saharan Africa, FSW constitutes one of the high-risk groups for HBV and STI acquisition and transmission. This is possibly sex workers have numerous sexual partners and they engage in unprotected sex and other forms of sex that cause contact with body fluids of a partner who may be infected with HBV [19].

According to 2019, Ethiopian demographic and health survey (EDHS) data of Ethiopia represents a stable, low-level, generalized epidemic with marked regional variations driven by most at-risk populations (MARPs). However, urban areas and females are more affected than rural areas and males [20]. Small towns are also becoming hot spots and can potentially bridge further the spread of HIV and HBV epidemic to rural settings by such population groups. Across the country, FSW carries a disproportionate burden of HIV and HBV [20]. A study conducted in Gondar and Mekele among FSW indicated the prevalence of HBV was 28.9 and 6%, respectively [11, 12]. These findings were greater than the findings from the Ethiopian general population 9% [21], from adult population in southwest Ethiopia 9% [22], and from a health professional in Gondar, 4.52% [23] and it can be evidenced as HBV infection is more prevalent among sex workers than the other population group.

Therefore, this study was proposed to determine the prevalence of Hepatitis B virus infection and its predisposing factors among FSWs in one of the hot spot cities of Ethiopia Hawassa. It could be important to emphasize the need for strengthening intervention strategies that address the risk and the marginalized group to stop the link or spread of HBV to the community through them and their sexual partners by designing vaccination schedules.

## Result

### Demographic and socio-economic conditions

Of the three hundred and eighty-four female sex workers who agreed to participate in the study, only three of them were excluded because of incomplete information from the questionnaire and also were not recruited for blood sample collection. For the remaining 381 FSWs, 45.7% of the participants were within the age group of 20–24 years with the mean (standard deviation [SD]) of age was 22.63 + 4.3 years. The total median and range age of the participants were 22 and 16–40 years. About 370(97.1%) were single and 11(2.9%) were previously married. Among 381 FSWs, 242(63.5%) respondents were from urban. According to their educational level, 306(80.3%) of the study participants were attended formal education. More than 35% of the study participants have a monthly income with the range of 1501 to 3000 Ethiopian Birr (Table 1).

**Table 1:** Socio-demographic characteristics of female sex workers at integrated services on Health Development project confidential clinic Hawassa 2019 (*n* = 381)

Variables		Frequency	Percent
Age in years	16–19	91	23.9
	20–24	174	45.7
	25–29	90	23.6
	30–34	17	4.5
	35–40	9	2.4
Marital status	Married	11	2.9
	Single	289	75.9
	Widowed	37	9.7
	Divorced	44	11.5
Educational Status	No formal education	75	19.7
	1–4	47	12.3
	5–8	204	53.5
	9–12	52	13.6
	Above 12th	3	.8
Residence	Rural	139	36.5
	Urban	242	63.5
Religion	Protestant	201	52.8
	Orthodox	164	43.0
	Muslim	7	1.8
	Catholic	9	2.4
Living status	Depend on family	295	77.4
	Had another job	75	19.7
	Other specify	11	2.9
Dependent people	Yes	187	49.1
	No	194	50.9
Dependent Size	No dependents	194	50.9
	1–2	141	37.0
	2–4	46	12.1
Average Monthly Income in ETB	501–1000	104	27.3
	1001–1500	87	22.8
	1501–3000	136	35.7
	3001–7000	54	14.2

*ETB* Ethiopian Birr

### Sexual and behavioral characteristics of female commercial sex workers

The overall prevalence of FSWs who were tested for HBV using ELISA (Enzyme linked Immunosorbent Assay) in the current study was 35(9.2%). Among 381 FSWs workers, 249 (65.4%) of them stayed for 2–5 years in sexual work. 240(63%) of them used condom consistently during sexual practice and 308(80.8%) had a habit of alcohol consumption. 100(26.2%) of them had a history of STI infection and 103(27%) of FSWs had a steady partner. The majority of 287 (73.3%) of the study subjects used vagina for sexual intercourse while 39(10.2%) of them used anal and vaginal, 55(14.4%) used oral and vaginal sexual practice. About 243 (63.8%) of the participants used injectable drugs and other stimulants to initiate their sexual desire (Table 2).

**Table 2:** Sexual behavior of female sex workers at integrated services on Health Development project confidential clinic Hawassa 2019 (*n* = 381)

Variables		Frequency	Percent
Place of sexual practice taken	Hotel based	172	45.1
	Street based	160	42.0
	Home based	48	12.6
	Any type	1	.3
Condom utilization during sex	Yes	240	63.0
	No	141	37.0
Frequency of condom utilization	Always	185	48.6
	Sometimes	43	11.3
	Rarely	12	3.1
Reason for not using condom	To satisfy customers	57	15.0
	To get more money	74	19.4
	Negligence	10	2.6
Breakage of condom during sex	Yes	67	17.6
	No	173	45.4
Action taken during breakage of condom.	Went to health facility	10	2.6
	Nothing	23	6.0
	Washing with water	34	8.9
Alcohol consumption	Yes	308	80.8
	No	73	19.2
Frequency of alcohol consumption	Always	74	19.4
	Sometimes having sex	156	40.9
	Rarely	78	20.5
Utilization of injectable drugs/ stimulants before sex	Yes	243	63.8
	No	138	36.2
Type of stimulants/injectable drugs	Khat only	162	42.5
	Khat and cigarette	15	3.9
	Khat and Shisha	40	10.5
	Shisha only	26	6.8
Duration of prostitute commitment	< 1 years	65	17.1
	2–5 years	249	65.4
	> 6 years	67	17.6
Sexual direction/position	vaginal sex only	287	75.3
	vaginal and anal	39	10.2
	vaginal and oral	55	14.4
HBV vaccination	Yes	1	.3
	No	380	99.7
History of STI infection	Yes	100	26.2
	No	281	73.8
Type of STI infection	Syphilis	18	4.7
	Gonorrhea	82	21.5
History of Genital ulcer	Yes	105	27.6
	No	276	72.4
Action on Genital ulcer	Went to a health facility	77	20.2
	treat with herbal treatment	23	6.0
	nothing did	5	1.3
Presence of steady partner	Yes	103	27.0
	No	278	73.0
Use of condom with steady partner	Yes	55	14.4
	No	48	12.6
Number of sexual partners per day	< 5	224	58.8
	> 5	157	41.2
Sex during menses	Yes	11	2.9
	No	370	97.1
A habit of sexual abuse/ harassment	Yes	44	11.5
	No	337	88.5
History of blood transfusion?	Yes	10	2.6
	No	371	97.4
Common use of Sharp material (tattooing)	Yes	30	7.9
	No	351	92.1
History of Abortion	Yes	36	9.4
	No	345	90.6
Place of abortion	Health facility	11	30.6
	Traditionally	25	69.4
HBV status	Positive	35	9.2
	Negative	346	90.8

*Key*: *HBV* Hepatitis B virus, *STI* Sexual transmitted infection

### Factors associated with HBV among female commercial sex workers

Many different variables were assessed for the presence or absence of association HBV with female sex workers using both bivariate and multivariate logistic regression models. The bivariate analysis was computed independently, and we have used a cut-off *P*-value of 0.2 to recruit and analyze the variables in the multivariate model.

In multivariate logistic regression analysis, FSWs who didn’t use condom commonly during sexual practice was significantly associated with acquiring of HBV (AOR = 6.38, CI 2.04–18.51), Condom breakage (AOR = 2.10, CI 1.95–4.65), use of stimulants, (AOR = 3.25, CI 1.59–18.63), history of STI, (AOR = 2.15, CI 1.02–6.93), history of genital ulcer, (AOR = 4.64, CI 1.31–11.35), number of clients used per day, (AOR = 3.25, CI 1.59–7.47), sex during menses (AOR = 5.85, CI (1.29–21.44), sexual assault (AOR = 2.93, CI 1.23–9.01), sharp material sharing, (AOR = 4.98, CI 1.34–10.95) and History of abortion, (AOR = 2.46, CI 1.18, 12.19), were also statistically associated with HBV infection at *P*-value < 0.05. Factors such as age, marital status, residence, alcohol consumption, and dependent size were not significantly associated with HBV infection among FSWs in the current study (Table 3).

**Table 3:** Factors associated with HBV among females sex workers at Hawassa City, Southern Ethiopia, 2019 (*n* = 381)

Variables		HBV (+) N (%)	HBV(−) N (%)	COR (95% CI)	***P***-value	AOR (95% CI)	***P***-value
Age in years	16–19	5(14.3)	86(24.6)	2.15(0.22, 20.73)	0.060	0.47(0.05, 4.49)	0.330
	20–24	16(45.7)	158(45.7)	1.23(0.15, 10.51)		0.81(0.09, 6.90)	
	25–29	10(28.6)	80(23.6)	1.0(0.11, 8.85)		1.0(0.11, 8.85)	
	30–34	3(8.6)	14(4.0)	0.58(0.05, 6.59)		1.71(0.15, 19.34)	
	35–40	1(2.9)	8(2.3)	1		1	
Marital status	Married	1(2.90)	10(2.9)	1	0.210	1	0.070
	Single	26(74.3)	263(76.0)	1.01 (0.13, 8.22)		1.00(0.10, 9.96)	
	Widowed	4(11.4)	33(9.5)	0.83(0.82,8.25)		1.01(0.34, 3.05)	
	Divorced	4(11.4)	40(11.6)	1.0(0.10, 9.96)		0.83(0.19, 3.55)	
Educational Status	No formal education	7(20.0)	68(19.7)	1.19(0.38, 3.76)	0.090	0.84(0.27, 2.66)	0.103
	1–4	3(8.6)	44(12.7)	1.79(0.42, 7.61)		0.56(0.13, 2.36)	
	5–8	19(54.3)	185(53.5)	1.19(0.45, 3.15)		0.84(0.32, 2.21)	
	9 and above	6(17.1)	49(14.2)	1		1	
Residence	Rural	5(14.3)	124(35.8)	1	0.015	1	0.022
	Urban	30(85.7)	222(64.2)	0.29(0.11, 0.79)		0.74(0.37, 1.51)	
Living status depend on	Family	31(88.6)	188(54.3)	1	0.001	0.98(0.43, 2.25)	0.140
	Not on family	4(11.4)	158(54.7)	6.51(2.25,18.85)		1	
Having dependent people	Yes	28(80.0)	159(46.0)	1	0.001	1	0.120
	No	7(20.0)	187(54.0)	4.70(2.00, 11.06)		1.28(0.43,3.84)*	
Monthly Income	501–1000	8(22.9)	90(26.0)	0.99(0.20, 2.39)	0.230		0.250
	1001–1500	15(42.9)	62(17.9)	0.69(0.08, 0.81)			
	1501–3000	8(22.9)	129(37.3)	0.25(0.29, 3.42)			
	3001–7000	4(11.4)	65(18.8)	1			
Dependent Size	No dependents	7(20.0)	187(54.0)	1	0.001	0.94(0.34, 2.61)	0.080
	1–2	23(65.7)	118(34.1)	0.19(0.80, 0.46)		0.55(0.17, 1.84)	
	2–4	5(14.3)	41(11.8)	0.31(0.09, 1.02)		1	
Condom use	Yes	10(28.6)	230(66.5)	1	0.001	1	0.010*
	No	25(71.4)	116(33.5)	4.96(2.30, 10.67)		6.38(2.04, 18.51)	
Condom Breakage during sex	Yes	6(60.0)	61(26.5)	4.16(1.13, 15.23)	0.030	2.10 (1.95,4.62)	0.030*
	No	4(40.0)	169(73.5)	1		1	
Alcohol consumption	Yes	21(60.0)	287(82.9)	3.24(1.56, 6.74)	0.001	1.21(0.45, 3.26)	0.140
	No	14(40.0)	59(17.1)	1		1	
Use of stimulant	Yes	29(82.9)	214(61.8)	2.98(1.21, 7.37)	0.013	3.25(1.59, 18.63)	0.018*
	No	6(17.1)	132(38.2)	1		1	
Duration of prostitution service	< 1	3(8.6)	62(17.9)	4.74(1.30, 17.34)	0.001	1	0.020*
	2–5	18(51.4)	223(64.5)	2.84(1.34, 6.04)		2.03(0.49, 8.50)	
	> 6	14(40.0)	61(17.6)	1		1.84(1.33, 2.14)	
Type of sex usually used	Vaginal only	24(68.6)	263(76.0)	1	0.080	1.20(0.34, 4.26)	0.980
	Vaginal and anal	5(14.3)	34(9.8)	0.62(0.22, 1.73)		0.75(0.29, 1.92)	
	Vaginal and oral	6(17.1)	49(14.2)	0.75(0.29, 1.92)		1	
Vaccinated	Yes	1(2.9)	1(0.3)	1	0.180	1	0.230
	No	34(97.1)	345(99.7)	10.15(0.62, 165.88)		0.99(0.06, 1.61)	
History of STI	Yes	20(57.1)	80(23.1)	4.43(2.17, 9.06)	0.001	2.15(1.02, 6.93)*	0.011*
	No	15(42.9)	266(76.9)	1		1	
History of Genital ulcer	Yes	18(51.4)	87(25.1)	3.15(1.56, 6.39)	0.001	4.64(1.31, 11.35)	0.004*
	No	17(48.6)	259(74.9)	1		1	
Number of clients used per day	< 5	9(25.7)	202(58.4)	1	0.001	1	0.018*
	> 5	26(74.3)	144(41.6)	4.05(1.84, 8.91)		3.25(1.59, 7.47)	
Sexes during menses	Yes	4(11.4)	7(2.0)	6.25(1.7, 22.53)	0.001	5.85(1.29,21.44)	0.002*
	No	31(88.6)	339(98.0)	1		1	
Sexual Assault	Yes	9(25.7)	35(10.1)	3.08(1.34, 7.09)	0.001	2.93(1.23,9.01)	0.007*
	No	26(74.3)	311(89.9)	1		1	
Blood received	Yes	2(5.7)	8(2.3)	1	0.120	1	0.080
	No	33(94.3)	338(97.7)	2.56(0.52, 12.56)		0.39(0.08, 1.92)	
Use of sharp material	Yes	8(22.9)	22(6.4)	4.36(1.78, 10.73)	0.001	4.98(1.34,10.95)	0.008*
	No	27(77.1)	324(93.6)	1		1	
History of Abortion	Yes	9(25.7)	30(8.7)	3.65(1.67, 8.49)	0.001	2.46(1.18, 12.19)	0.001*
	No	26(74.3)	316(91.3)	1		1	

*Key*: *AOR* adjusted odds ratio, *CI* confidence interval, *COR* crude odds ratio, *OR* odds ratio, *STI* Sexually Transmitted infections

* = Significant at *p* < 0.005

## Discussion

Ethiopia has been classified as an HBV endemic zones [22]. Although this classification gives a fair picture of the global HBV endemicity, it fails to take into account the variability of the disease within various population groups [22]. Also, most information on HBV prevalence in Ethiopia is available from blood donors and pregnant women [23]. In this study, the prevalence of HBsAg marker (indicating HBV infection) among female sex workers at integrated services on Health Development project confidential clinic Hawassa was 9.2% (CI:95%, 6.3–12.1%).

This prevalence is higher than in many other population groups studied in Ethiopia. This is critical owning to the fact that this group has a greater probability of transmitting and maintaining the virus in the community.

This finding was lower than the study reported from Gondar Ethiopia (11.9%) [24], Nigeria (17.1%) [ (25)], Cameroon (36%) [26], Argentina (14.4%) [27], Shanghai, China 12.3% [28] in two different studies in Brazil 17.1% [29] and 23.1% [30].. The current finding was higher than the study conducted in Mekelle, Ethiopia (6%) [31], Iran (1.2%) [32], Rwanda (2.5%) [33], Congo (7.3%) [34], Italy (3.5%) [35], Venezuela (3.8%) [36], and Afghanistan (6.54%) [37]. This difference might be due to the difference between diagnostic tools, sample size, differences in socio-demographic and socio-economic environments. Furthermore, the difference in the prevalence estimates is also likely to be influenced by stigma and discrimination and also study settings.

Compared to the prevalence general population (6%) [31] it can be assumed that CSWs were more likely to have a high prevalence (9.2%) of HBV in this study. Comparing this study results with the general population indicates that there less emphasis on this group of population.

The prevalence of HBV according to the age group of FSWs the highest prevalence 16(45.7%) was accounted within the age group of 20–24 years. But, the finding was not statistically significant (*p* > 0.05) which was inconsistent with the study conducted in Nigeria [25]. On the other hand, a similar finding to the current study was reported from Ethiopia, Dessie [13], and Burkina Faso [8]. The possible reasons may be the early onset of sexual intercourse represents an increased risk for sexually transmitted infections.

In this study, educational status, marital status, monthly income, vaccination, and alcohol consumption were not statically significant association with HBV similar to other studies conducted in three Afghan cities among female sex workers [38] in Mekelle among commercial sex workers [31] and in Tehran, Iran among female sex workers [39].

Sexual intercourse during menses is statically associated with acquiring HBV infection in sex workers (AOR = 5.85, 95% CI: 1.29–21.44), FSW who have a history of abortion (AOR = 2.46,95% CI:1.18,12.19) and a genital ulcer (AOR = 4.64, 95% CI:1.31,11.35) were 24 and 46 times more risk full than those who have not a history of abortion and genital ulcer. Similar findings were reported from Mekelle, Ethiopia [31], Brazil [40], and Nigeria [25].

Sexual assault was 12 times more exposed for acquiring HBV (AOR = 2.93, 95% CI:1.23,9.01) similar findings were reported from Iran [38] and Nigeria [25]. Number of sexual partners (AOR = 3.25, 95% CI: 1.59, 7.47), duration on sex work (AOR = 1.84, 95% CI: 1.33, 2.14) and condom use (AOR = 6.38, 95% CI: 2.04, 18.51). In line with our finding was reported from Mexico [41]. The possible explanation may be multiple clients produce greater vulnerability to risks for low adherence to the use of condoms in all sexual relations.

Limitations of the current study were due to significant difficulties encountered in attempting to recruit the needed sample size from the FSWs population. Female sex workers in Ethiopia are mainly street-based which makes it quite tough and threatening to reach them. A follow-up study with a larger sample size with longitudinal study in the different study areas is worthwhile to add to the literature.

## Conclusion

Despite the limitations in difficulties encountered in attempting to recruit the study participants, the prevalence of HBV infection among FSWs was relatively high compared to the general population in the current study. Furthermore, different factors like sociodemographic, behavioral, clinical, and previous history-related information have been also assessed for the presence of association with HBV infection. Condom use, history of genital ulcer, sexes during menses, sexual assault, history of abortion, Number of sexual partners, duration of sex work, and sharing of sharp material were highly associated with HBV infection among female sex workers. Meanwhile, they are at a higher risk of acquiring HBV infection, as indicated above due to high-risk behaviors of sex practice and lack of successful HBV immunization evidence. Preparedness should be initiated to prevent the potential risk of HBV infection. Since they can be a source of infection for the community, first, a mass screening activity or a longitudinal survey study on FSWs should be done. Then, a preventive approach and appropriate treatment scheme for HBV should be developed.

Finally, the government, any other nongovernmental organizations, civic society, and religious institutions should work together to alleviate the problem by counseling and recruiting them on other productive job sectors that are found in the country.

## Methods

### Study area

This study was conducted in Sidama regional state of Hawassa City in one of the nongovernmental institutions founded to support marginalized populations to give integrated services on Health Development (ISHDO) project confidential clinic. Hawassa city is the central town of the regional state located 270 km from Addis Ababa at the shores of Lake Hawassa in the Great Rift Valley area of southern Ethiopia. It is known by diverse cultural constituents, socio-economic benefit, and good tourist destination and recreational city with a total population of 302, 000 according to the Worldometer report of 2021 [21].

### Study design, period, and population

A cross-sectional study design was employed among adolescent and young adult female sex workers from July 1–November 31st, 2019, who are living in Hawassa city working as a commercial sex worker for at least 3 months and registered in the nongovernmental institutions founded to support marginalized populations to give at integrated services on Health Development (ISHDO) project confidential clinic were selected and included in the study.

A single population proportion formula was used to estimate the sample size, following the assumption to consider: 95% confidence interval (*Zα*/2 = 1*:*96), 50% proportion was taken due to limited data about HBV among FSWs in the area and nearby localities, and 5% margin of error.

$$\boldsymbol{n}={\left(\boldsymbol{Za}/\mathbf{2}\right)}^{\mathbf{2}}\;\mathbf{P}\;\left(\mathbf{1}\hbox{-} \mathbf{P}\right)/{\mathbf{d}}^{\mathbf{2}}=\mathbf{384}$$

A total of 384 female sex workers were included from eight sub-cities and from all kebeles who were pre-registered in the registration book. They were selected using a simple random sampling method by using assigned ex-sex workers, namely: “Demand creator” and preregistered FSWs to bring their counterparts by moving from home to home and brought to this special clinic were participated.

Female sex workers registered in the nongovernmental institutions founded to support marginalized populations to give an integrated service on Health Development (ISHDO) project confidential clinic were included in the study. We have considered and defined women as FSWs when they are living and commercializing sex for the last 3 months in Hawassa City. Female sex workers with age greater than or equal to 18 years old and who are willing to participate in this study were included, but those FSWs with apparent mental or physical illness that limit them from an interview and those who are not available during the study period were excluded.

### Specimen collection

A simple random sampling technique was used to select 384 study participants during the study period. Before the actual data collection, we have studied the average number of FSWs registered in the clinic. About 626 FSWs were registered in the clinic at start of data collection including the newly registered participants with daily visit of 18 FSWs. Every 2nd participant was going to be included in the study each day.

#### Data collection methods

After obtaining an informed and written consent, a standardized questionnaire was used to collect the sociodemographic, behavioral, and other predisposing variables that are associated with the dependent variable. Five milliliters of venous blood was drawn under aseptic conditions by trained data collectors. The sample was labeled and processed by centrifugation at 3500 rpm for 5 min to obtained serum and stored at -20^0^c in the refrigerator until it was tested. HBsAg was detected from serum samples by using AiD™ antibody sandwich HBsAg ELISA method (WANTAI HBV diagnostics AiD™ HBsAg ELISA). The test was conducted following the manufacturer’s instructions and the Microplates read at a wavelength of 450 nm using the Enzyme-linked immune assay (ELISA) reader. The presence or absence of HBsAg was determined by relating the absorbance of the unknown sample to the cut-off value.

### Statistical analysis

Data were cleaned and checked and entered into SPSS version 21 for analysis. The data were analyzed using descriptive summary using frequencies, appropriate summary tables, and cross tabs, and relevant information was summarized to present results. Bivariate logistic regression analysis was performed to identify the factors associated with HBV infection. Variables having a *P*-value of < 0.2 in bivariate analysis were eligible for multivariate logistic regression analysis to control potential confounding factors. A *p*-value of less than 0.05 is considered as statistical significance.

## Data Availability

The datasets used and/or analyzed during the current study are available from the corresponding author on reasonable request.

## Abbreviations

ISHDO: Integrated services on Health development
HBV: Hepatitis B virus
HBsAg: Hepatitis B surface antigen
FSW: Female sex workers
STIs: Sexually transmitted infections
AOR: Adjusted odds ratio
COR: Crude odds ratio
WHO: World Health Organization
